# Association between maintenance dialysis and tuberculosis treatment outcomes in Amazonas, Brazil: a population-based record-linkage cohort study (2001–2023): Maintenance Dialysis and Tuberculosis Outcomes

**DOI:** 10.1186/s12879-026-13991-3

**Published:** 2026-07-13

**Authors:** Samanta Samara Bicharra dos Santos, Djane Clarys Baia-da-Silva, Jair dos, Santos Pinheiro, Leandro Sousa Garcia, Renata Spener-Gomes, Lara Bezerra de Oliveira de Assis, Isabella Bonifácio, Brige Ferreira, Beatriz Barreto-Duarte, Moreno M. S. Rodrigues, Bruno B. Andrade, Rodrigo Carvalho de Menezes, Marcelo Cordeiro-Santos

**Affiliations:** 1 Universidade do Estado do Amazonas. Programa de Pós-graduação em Medicina Tropical, Manaus, Brazil; 2 Fundação de Medicina Tropical Dr. Heitor Vieira Dourado, Manaus, Brazil; 3 Hospital Universitário Getúlio Vargas, Manaus, Brazil; 4 Fundação de Vigilância em Saúde do Amazonas Dra. Rosemary Costa Pinto, Manaus, Brazil; 5 Instituto Lêonidas & Maria Deanne, Manaus, Brazil; 6 Universidade Nilton Lins, Manaus, Brazil; 7 Universidade Federal do Amazonas, Manaus, Brazil; 8 Universidade Federal de Santa Catarina, Programa de Pós-Graduação em Enfermagem, Florianópolis, Brazil; 9 Laboratório de Pesquisa Clínica e Translacional, Instituto Gonçalo Moniz, Salvador, Bahia Brazil; 10 Pós-graduação em Medicina e Saúde Humana, Escola Bahiana de Medicina e Saúde Pública, Salvador, Brazil; 11 Instituto de Pesquisa Clínica e Translacional, Faculdade ZARNS, Salvador, Bahia Brazil; 12 Fundação de Medicina Tropical Dr. Heitor Vieira Dourado (FMT-HVD), Av. Pedro Teixeira, 25, Dom Pedro, Manaus, Amazonas 69040-000 Brazil

**Keywords:** Tuberculosis, Maintenance dialysis, Kidney failure, Treatment outcomes, Directly observed therapy, Brazil

## Abstract

**Background:**

Although maintenance dialysis (MD) affects only a small proportion of patients with tuberculosis (TB), individuals receiving dialysis are at increased risk of TB and may be more likely to experience unfavourable treatment outcomes. Evidence on this association remains limited in Brazil, particularly in high-burden settings characterized by geographic barriers and challenges in access to specialized healthcare services. We assessed the association between MD and unfavourable TB treatment outcomes in Amazonas, Brazil.

**Methods:**

We conducted a retrospective population-based cohort study including all new TB cases reported in Amazonas, Brazil, between 2001 and 2023. Probabilistic record linkage was performed between the state TB surveillance system (SINAN-TB) and a dialysis registry to identify patients receiving MD before TB diagnosis. The primary exposure was MD prior to TB diagnosis. Unfavourable outcomes comprised death from any cause, loss to follow-up, or treatment failure. Multivariable logistic regression was used to estimate adjusted odds ratios (aORs) with 95% confidence intervals (CIs).

**Results:**

Among 58,611 TB cases included in the analysis, 112 (0.2%) had evidence of MD before TB diagnosis. MD was strongly associated with higher odds of unfavourable outcomes (aOR 5.45; 95% CI 3.60–8.24). Illicit drug use (aOR 2.18; 95% CI 1.97–2.40), HIV/AIDS (aOR 2.06; 95% CI 1.94–2.19), mental illness (aOR 1.53; 95% CI 1.27–1.83), alcohol use (aOR 1.38; 95% CI 1.29–1.48), and smoking (aOR 1.30; 95% CI 1.20–1.42) were also associated with increased odds of unfavourable outcomes. In contrast, directly observed therapy (DOT) (aOR 0.67; 95% CI 0.63–0.71), female sex (aOR 0.82; 95% CI 0.79–0.85), and diabetes mellitus (aOR 0.84; 95% CI 0.78–0.91) were associated with reduced odds.

**Conclusions:**

Within a large population-based TB cohort, MD was a rare but clinically important exposure that was strongly associated with unfavourable treatment outcomes. The protective effect of DOT highlights a feasible intervention pathway. These findings support the integration of TB care into dialysis services and reinforce the need to prioritize this population for targeted TB screening and preventive strategies in high-burden settings.

**Clinical trial number:**

Not applicable.

**Supplementary Information:**

The online version contains supplementary material available at https://doi.org/10.1186/s12879-026-13991-3.

## Background

Tuberculosis (TB) remains a leading cause of global illness and death, affecting 10.7 million people and causing 1.2 million deaths among HIV-negative individuals in 2023 [1]. The disease burden is particularly high in low- and middle-income countries such as Brazil [2, 3]. In Brazil, the state of Amazonas faces a critical epidemiological situation, with an annual incidence of 94.7 cases per 100,000 people and mortality rates that are much higher than the national average (5.1 compared to 2.8 deaths per 100,000) [4]. This gap is exacerbated by geographic and socioeconomic obstacles specific to the Amazon region, such as the isolation of rural communities, poor transportation infrastructure, and limited access to healthcare services, factors that delay diagnosis and lead to poorer health outcomes [5]. These challenges are particularly relevant for patients on maintenance dialysis (MD). In Amazonas, renal replacement therapy is highly centralized, with almost all dialysis services concentrated in the capital, Manaus. Individuals from distant municipalities often face long commutes and depend on referral systems to access specialized nephrology care. Simultaneously, TB control actions and dialysis services are generally organized within separate structures, with little integration across screening, surveillance, and treatment activities. This fragmentation can lead to delays in diagnosis, treatment initiation, and continuity of care among vulnerable patients affected by both conditions.

Systematic screening of high-risk populations is essential for TB control [6]. Among these groups, individuals with chronic kidney disease (CKD), especially those on dialysis, face a significantly higher risk of developing active TB due to dialysis-related immunosuppression [6, 7]. Several biological mechanisms have been proposed to explain this increased susceptibility. Advanced kidney disease and MD are associated with uremia-related immune dysfunction, chronic systemic inflammation, impaired cellular immunity, nutritional deficiencies, and alterations in both innate and adaptive immune responses [8–12]. Together, these factors may increase vulnerability to *Mycobacterium tuberculosis* infection and contribute to poorer clinical outcomes once active disease develops [6, 13, 14]. Meta-analytic data show that dialysis populations have a 3.6 times higher rate of active TB compared to the general population [15]. However, although the increased incidence is well-documented, the relationship between MD and TB treatment outcomes remains poorly understood, particularly in high-burden settings.

Quantifying this relationship in the Brazilian Amazon has historically been constrained by reliance on passive TB reporting systems, such as the TB national health information system (SINAN-TB), which lack reliable linkage to specific CKD registries [16]. This fragmentation of health data obscures the true impact of renal comorbidities on TB outcomes. To address this gap, we used probabilistic record linkage between the SINAN-TB and a comprehensive dialysis database covering nearly all patients receiving MD in Amazonas, Brazil. This study used a population-based cohort of TB patients, integrated into a comprehensive dialysis registry, to assess whether MD is associated with adverse TB treatment outcomes, defined as death from any cause, loss to follow-up, or treatment failure, in Amazonas, Brazil. Furthermore, we explored demographic, clinical, and behavioral factors that may influence treatment outcomes in this high-prevalence setting.

## Methods

### Study design and setting

We conducted a retrospective, population-based cohort study that included all reported TB cases in Amazonas between 2001 and 2023. MD was the main exposure variable, assessed via a probabilistic linkage to the state dialysis registry. The study population comprised all new cases of TB reported in the SINAN-TB of the Amazonas, Brazil, with a diagnostic date between January 2001 and December 2023 and people with chronic kidney disease who were actively registered or who died by December 2023, with a MD start date from 1994 onwards, in five of the six private clinics specializing in renal replacement therapy, affiliated with the Public Health System of the state of Amazonas, located in the state capital, Manaus. These clinics are the referral centers for dialysis services in the state, as there are no public institutions that provide this service. All patients registered at these institutions were included, regardless of dialysis modality, including those undergoing hemodialysis (*n* = 2,002; 62.2%) or peritoneal dialysis (*n* = 32; 1.0%), and 1,185 (36.8%) with modality not informed, as well as those in active treatment, transferred, transplanted, or deceased during the study period. One dialysis center did not participate in the study. Although the participating clinics account for most renal replacement therapy services available in Amazonas, some patients receiving dialysis outside the participating centers may not have been identified, potentially resulting in underascertainment of exposed individuals.

Our setting was selected because it has a substantially higher TB incidence than the Brazilian national average. For this study, new TB cases were defined as individuals notified in SINAN-TB with a diagnosis of TB, including pulmonary, extrapulmonary, and mixed forms, irrespective of microbiological confirmation.

### Data sources

Data were obtained from two sources. First, SINAN-TB was used to identify notified TB cases and extract sociodemographic, clinical, laboratory, and treatment outcome data. Authorization for data access was granted by the Amazonas Health Surveillance Foundation (FVS-AM). Second, an individual-level registry of patients receiving MD through December 2023 was compiled from five private clinics affiliated with the Public Health System of the state of Amazonas, which authorized participation in the study. The databases were updated in August 2024.

### Probabilistic record linkage

Probabilistic record linkage was performed between SINAN-TB and the clinic-based dialysis registry to identify TB cases with a matched dialysis record. The linkage algorithm employed approximate string matching based on three primary identifiers: patient name, mother’s name, and date of birth. An initial automated matching step used approximate string matching based on Levenshtein distance, with a minimum similarity threshold of approximately 80% to generate candidate pairs. All candidate matches were subsequently reviewed manually to assess plausibility and confirm concordance across the identifying variables. Records judged to represent homonyms, insufficient similarity, or inconsistent matches were excluded.

### Variable, exposure, and case definitions

Variable definitions followed the official criteria established by SINAN-TB and the Manual of Recommendations for TB Control in Brazi [17]. Detailed variable definitions and coding procedures are available in the SINAN-TB data dictionary, which served as the reference for data extraction and classification [18]. Race/color was classified according to the Brazilian Institute of Geography and Statistics (IBGE) categories (White, Black, Brown/Pardo [mixed], Yellow [Asian], and Indigenous). A peri-urban area is a region at the interface between urban and rural zones, typically characterized by mixed infrastructure and limited access to healthcare services.

The primary exposure was MD prior to TB diagnosis. Exposure status was determined after probabilistic record linkage between SINAN-TB and the dialysis registry, followed by temporal classification based on the date of MD initiation relative to the TB diagnosis. Renal function measurements (e.g., eGFR) were not used to define exposure, as classification was based exclusively on documented MD.”

CKD cases were identified using two complementary approaches. First, CKD was identified by analyzing the open-text field (“agravoutde”) in the SINAN-TB database, which is completed by healthcare professionals to record comorbidities not captured in predefined coded variables. Second, CKD cases were identified through probabilistic linkage with a registry of patients receiving care at specialized dialysis centers.

All TB cases identified through either source were initially classified as having CKD. These cases were subsequently evaluated to determine the temporal relationship among CKD, dialysis initiation, and TB diagnosis. Individuals were classified as exposed only if there was evidence of MD initiated before TB diagnosis, regardless of whether this information was obtained from the SINAN-TB open-text field or dialysis registry.

**Fig. 1:**
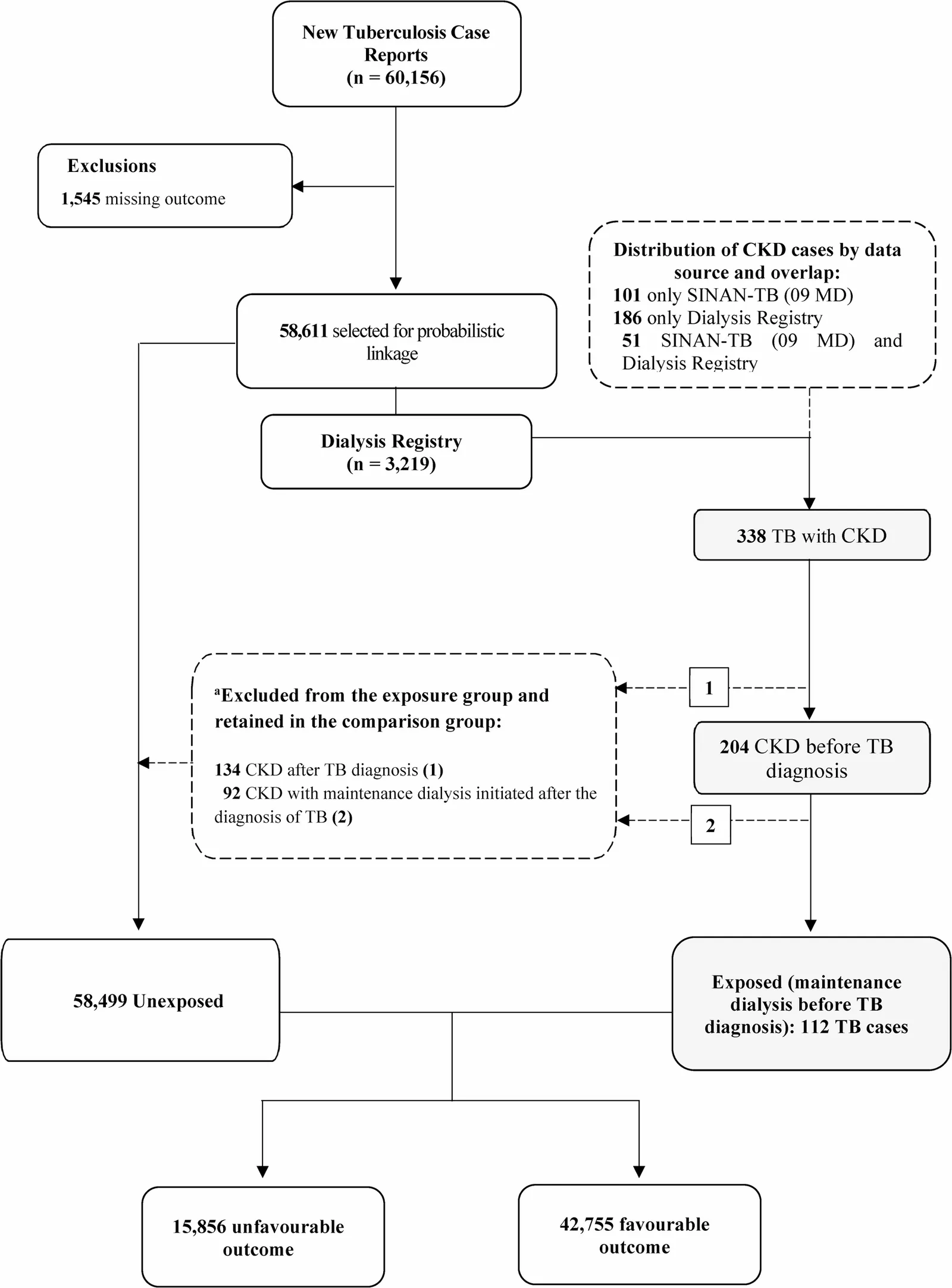
Flowchart of probabilistic record linkage and temporal classification of maintenance dialysis exposure among tuberculosis cases, Amazonas, Brazil. The flowchart illustrates the identification of the analytic cohort from the SINAN-TB database, the probabilistic linkage to the dialysis registry, and the stepwise classification of chronic kidney disease (CKD) and maintenance dialysis (MD) exposure based on the temporal relationship to TB diagnosis. Among 338 matched TB cases with CKD, 204 had CKD documented before TB diagnosis; of these, 112 had evidence of MD initiated prior to TB diagnosis and were classified as exposed. Individuals with CKD diagnosed after TB or with initiation of MD after TB diagnosis were excluded from the exposure group and retained in the comparison group. Source: SINAN-TB/FVS-RCP; Registry of CKD cases – MD, Amazonas, Brazil, 2001–2023 (updated August 2024). Abbreviations: TB, tuberculosis; CKD, chronic kidney disease; MD, maintenance dialysis; SINAN-TB, Brazil’s national TB surveillance system. Notes: Exposure classification was based on the date of initiation of MD in relation to the date of TB diagnosis

Cases with CKD diagnosed after TB or with initiation of MD after TB diagnosis were excluded from the exposure group and retained in the comparison group. This classification process is illustrated in Fig. 1.

### Clinical outcomes

The primary outcome was the TB treatment outcome, categorized as favourable and unfavourable. A favourable outcome was defined as a cure. An unfavourable outcome was defined as a composite endpoint including loss to follow-up, death, or treatment failure.

Loss to follow-up comprised treatment interruption or ≥ 30 consecutive days, as well as primary non-compliance, defined as failure to initiate treatment after diagnosis. Transfer cases were classified as loss to follow-up when there was no information on the treatment outcome at the destination unit, following the procedures established by SINAN-TB, which, operationally, equates these situations to therapeutic interruptions. Transfers were also categorized by type (within the same municipality, within the same state, to another state, or internationally), although this information was not always available in the initial versions of the records. Given the low frequency of this case profile in the analyzed set, it was concluded that such classification would have a negligible impact on outcome estimates.

Death included both deaths attributed to TB and deaths due to other causes occurring during treatment, as recorded in the SINAN-TB outcome classification. Treatment failure included persistent bacteriological positivity at the end of treatment, development of drug-resistant TB, or major treatment modification because of adverse events or toxicity. Cause-of-death information was obtained from routine surveillance records and could not be independently validated. In addition, some transferred patients may have been misclassified as loss to follow-up if treatment outcomes were not updated at the receiving facility.

### Statistical analysis

Continuous variables were summarized using medians and interquartile ranges (IQRs), while categorical variables were presented as absolute frequencies and percentages. Between-group comparisons were performed using the Mann-Whitney U test for continuous variables and the chi-square test or Fisher’s exact test for categorical variables, with a significance level of *p* < 0.05.

The primary exposure of interest was MD. Given that MD is, by definition, a marker of chronic kidney disease (since all dialysis patients had a prior diagnosis of CKD before TB), only MD, as the exposure of interest, was included as a renal variable in the model. Therefore, CKD was not included as an independent covariate to avoid collinearity between variables.

Multivariable logistic regression was used to estimate the independent association between MD and unfavourable TB treatment outcomes. Other sociodemographic, clinical, and behavioral variables were included as potential confounders and retained according to predefined statistical criteria. Other covariates were selected using a backward stepwise approach. The threshold of *p* < 0.20 was adopted to avoid premature exclusion of potentially important variables during the screening stage, whereas *p* < 0.05 was used for retention in the final model to identify variables independently associated with the outcome after adjustment for potential confounders.

All analyses were conducted using R software (R Foundation for Statistical Computing, Vienna, Austria).

### Ethical approval statement

This study was conducted in accordance with the ethical principles outlined in the Declaration of Helsinki and the Brazilian National Health Council Resolution No. 466/2012. The study protocol was approved by the Ethics Committee of the Fundação de Medicina Tropical (CAAE: 74993423.8.0000.0005). The ethics committee waived the requirement for written informed consent due to the study’s retrospective nature and the use of anonymized secondary data.

## Results

A total of 60,156 new TB cases were identified in the SINAN-TB database. After excluding 1,545 records (2.6%) without documented treatment outcomes, 58,611 cases (97.4%) were included in the analytic cohort (Fig. 1). Probabilistic linkage with the dialysis registry (3,219) identified 338 matched TB cases (0.6%) with CKD and 60% of these (*n* = 204) had CKD prior to TB diagnosis. A total of 112 (33.1% of matched records; 0.2% of the analytic cohort) had evidence of MD before TB diagnosis, and 58,499 individuals had no evidence of MD prior to TB diagnosis (exposed-to-unexposed 1:522). Overall, 15,856 patients (27.1%) experienced an unfavourable treatment outcome.

The cohort was predominantly male (60.5%), with a median age of 35.2 years (IQR 24.1–51.1), and 27.1% experienced an unfavourable treatment outcome. Male sex, residence in Manaus, behavioural factors (illicit drug use, smoking, and alcohol consumption), and comorbidities such as HIV/AIDS and CKD were more frequent among individuals with unfavourable outcomes (all *p* < 0.001). MD, although rare (0.2%), was disproportionately represented among patients with unfavourable outcome (0.5% vs. 0.1%; *p* < 0.001) (Table 1). Patients receiving MD experienced a substantially higher proportion of unfavourable treatment outcomes compared with those not receiving dialysis (73 (65.2%) versus 15,783 (27.0%), *p* < 0.001) (Supplementary Table S1).

**Table 1:** Sociodemographic and clinical characteristics of tuberculosis patients according to treatment outcomes, Amazonas, Brazil, 2001–2023

Characteristics	Study population (*n* = 58,611)	Favourable (*n* = 42,755)	Unfavourable (*n* = 15,856)	*P*-value	Completeness^b^
**Sex**,** male**	35,458 (60.5%)	24,942 (58.3%)	10,516 (66.3%)	< 0.001	100%
**Region**,** Manaus**	47,559 (81.1%)	33,932 (79.4%)	13,627 (85.9%)	< 0.001	100%
**Age**,** Years**	35.2 [24.1;51.1]	34.9 [23.7;50.5]	36.2 [25.2;53.1]	< 0.001	98.9%
**Race**				< 0.001	92.6%
White	5706 (10.5%)	4328 (11.0%)	1378 (9.25%)		
Black	1826 (3.4%)	1283 (3.3%)	543 (3.7%)		
Yellow	360 (0.7%)	272 (0.7%)	88 (0.6%)		
Brown	42,895 (79.1%)	30,810 (78.3%)	12,085 (81.1%)		
Indigenous	3466 (6.4%)	2664 (6.8%)	802 (5.4%)		
**Zone**				0.520	98.3%
Urban	51,386 (89.2%)	37,554 (89.2%)	13,832 (89.2%)		
Rural	6018 (10.4%)	4397 (10.4%)	1621 (10.5%)		
Peri-urban	186 (0.3%)	129 (0.3%)	57 (0.4%)		
**Chest radiography**				< 0.001	100%
Normal	15,578 (26.6%)	11,566 (27.1%)	4012 (25.3%)		
Suggestive of TB	42,690 (72.8%)	30,973 (72.4%)	11,717 (73.9%)		
Other	343 (0.6%)	216 (0.5%)	127 (0.8%)		
**Microbiologic diagnosis**	39,550 (67.5%)	29,292 (68.5%)	10,258 (64.7%)	< 0.001	100%
**Contacts**	3.0 [1.0;5.0]	3.0 [2.0;5.0]	2.0 [1.0;5.0]	< 0.001	79.3%
**TB type**				< 0.001	100%
Pulmonary	48,875 (83.4%)	36,107 (84.5%)	12,768 (80.5%)		
Extrapulmonary	7867 (13.4%)	5574 (13.0%)	2293 (14.5%)		
Pulmonary and Extrapulmonary	1869 (3.2%)	1074 (2.5%)	795 (5.0%)		
**Pleural or lymph node TB**	4538 (7.7%)	3332 (7.8%)	1206 (7.6%)	0.451	100%
**Comorbidities**					
PLWHA^a^	5487 (9.4%)	2929 (6.8%)	2558 (16.1%)	< 0.001	100%
CKD	338 (0.6%)	171 (0.4%)	167 (1.1%)	< 0.001	69.8%
CKD – before TB diagnosis	204 (0.4%)	61 (0.1%)	143 (0.9%)	< 0.001	50.0%
Maintenance dialysis	112 (0.2%)	39 (0.1%)	73 (0.5%)	< 0.001	100%
Diabetes Mellitus	3977 (6.8%)	2946 (6.9%)	1031 (6.5%)	0.097	100%
Mental illness	548 (0.9%)	336 (0.8%)	212 (1.3%)	< 0.001	100%
**Consumption habits**					
Drug use	2357 (4.0%)	1086 (2.5%)	1271 (8.0%)	< 0.001	100%
Smoking	3493 (5.9%)	1917 (4.5%)	1576 (9.9%)	< 0.001	100%
Alcohol	5144 (8.8%)	2980 (6.9%)	2164 (13.6%)	< 0.001	100%
**DOT**	11,100 (18.9%)	8938 (20.9%)	2162 (13.6%)	< 0.001	100%

Source: SINAN-TB/FVS-RCP; Registry of CKD cases – Maintenance Dialysis, Amazonas, Brazil, 2001–2023 (updated August 2024)

Abbreviations: TB, Tuberculosis; PLWHA, People living with HIV/AIDS; DOT, Directly Observed Treatment; CKD – Chronic kidney disease

Notes: a They represented 94.4% of all HIV infection cases

b Completeness was assessed as the proportion of records with valid information in each corresponding field of the notification form. For Comorbidities and Consumption Habits, all records with reported conditions were considered complete. For CKD, completeness was defined based on the availability of a recorded date of initiation of maintenance dialysis

To better characterize the exposure, baseline characteristics were compared between patients with and without MD (Supplementary Table S1). Patients receiving MD were older (median 48.6 vs. 35.2 years; *p* < 0.001), more likely to reside in Manaus (99.1% vs. 81.1%; *p* < 0.001), had lower rates of microbiological confirmation (50.9% vs. 67.5%; *p* < 0.001), and more frequent pleural or lymph node involvement (26.8% vs. 7.7%; *p* < 0.001). Behavioural risk factors did not differ between groups.

**Fig. 2:**
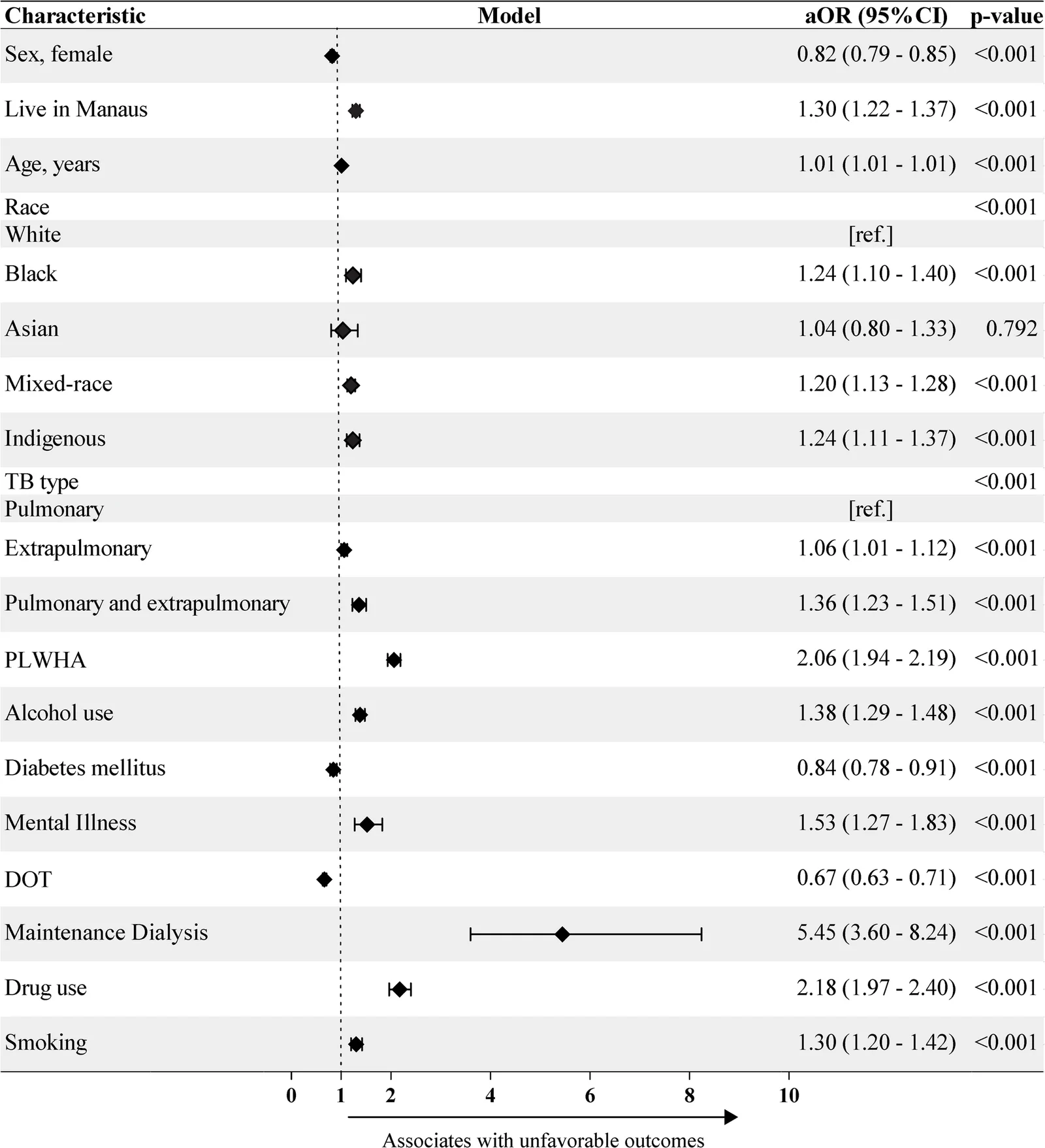
Multivariable logistic regression analysis of factors associated with unfavourable treatment outcomes among tuberculosis (TB) patients, Amazonas, Brazil, 2001–2023. The figure presents adjusted odds ratios (aORs) and 95% confidence intervals (CIs) from a multivariable logistic regression model assessing factors associated with unfavourable TB treatment outcomes. Maintenance dialysis (MD) prior to TB diagnosis was associated with higher odds of unfavourable outcomes (aOR 5.45; 95% CI 3.60–8.24). Other factors associated with increased odds included illicit drug use (aOR 2.18; 95% CI 1.97–2.40), HIV/AIDS (aOR 2.06; 95% CI 1.94–2.19), mental illness (aOR 1.53; 95% CI 1.27–1.83), alcohol use (aOR 1.38; 95% CI 1.29–1.48), smoking (aOR 1.30; 95% CI 1.20–1.42), and residence in Manaus (aOR 1.30; 95% CI 1.22–1.37). Female sex (aOR 0.82; 95% CI 0.79–0.85), directly observed therapy (DOT) (aOR 0.67; 95% CI 0.63–0.71), and diabetes mellitus (aOR 0.84; 95% CI 0.78–0.91) were associated with reduced odds of unfavourable outcomes. The vertical reference line indicates an odds ratio of 1. Values greater than 1 indicate increased odds of unfavourable outcomes, whereas values less than 1 indicate reduced odds. Abbreviations: TB, tuberculosis; aOR, adjusted odds ratio; CI, confidence interval; DOT, directly observed therapy; PLWHA, people living with HIV/AIDS. Notes: The model was adjusted for sociodemographic, clinical, and behavioural variables included in the multivariable analysis. Reference categories are indicated in the figure

In the adjusted model, MD was independently associated with higher odds of unfavourable outcomes (aOR 5.45; 95% CI 3.60–8.24; *p* < 0.001). Increased odds were also observed for illicit drug use (aOR 2.18; 95% CI 1.97–2.40), HIV/AIDS (aOR 2.06; 95% CI 1.94–2.19), mental illness (aOR 1.53; 95% CI 1.27–1.83), alcohol use (aOR 1.38; 95% CI 1.29–1.48), smoking (aOR 1.30; 95% CI 1.20–1.42), and residence in Manaus (aOR 1.30; 95% CI 1.22–1.37). Female sex (aOR 0.82; 95% CI 0.79–0.85), directly observed therapy (DOT) (aOR 0.67; 95% CI 0.63–0.71), and diabetes mellitus (aOR 0.84; 95% CI 0.78–0.91) were associated with reduced odds (all *p* < 0.001) (Fig. 2), although this finding should be interpreted cautiously given its inconsistency with the broader literature.

## Discussion

In this large population-based cohort study on TB, MD was a rare exposure, present in only 0.2% of cases, but showed a strong association with unfavorable treatment outcomes. This result demonstrates that even infrequent comorbid conditions can identify important clinical subgroups in which the risk of adverse outcomes is considerably higher. In the adjusted analysis, patients with a matched record indicating MD had more than fivefold higher odds of unfavourable outcomes than TB patients without a matched dialysis-registry record. DOT was associated with lower odds of unfavourable outcomes. In the adjusted model, surprisingly, diabetes mellitus showed an inverse association with unfavorable tuberculosis treatment outcomes, a result that diverges from the usual findings in the literature. This finding is analyzed with caution, as will be discussed below.

Although the associations identified in this study are robust, it is essential to interpret them in light of the existing body of evidence. CKD, particularly in advanced stages and among patients receiving MD, is a well-established risk factor for both increased TB incidence and unfavourable clinical outcomes [6, 15, 19–26]. This relationship extends across the spectrum of renal dysfunction, suggesting a progressive impact of kidney disease on TB susceptibility and prognosis [6, 15, 19–26]. These alterations increase susceptibility to *M. tuberculosis* infection and may compromise the host response after disease onset. In addition to this biological vulnerability, patients with advanced CKD frequently present with atypical clinical manifestations of TB, including a higher frequency of extrapulmonary disease and nonspecific symptoms, which can delay diagnosis and complicate clinical management [6, 15, 19, 22, 25, 26]. Patients on MD included in our study had a higher frequency of pleural and lymph node involvement and a reduced rate of microbiological confirmation, consistent with these observations. These characteristics may favor increased diagnostic uncertainty and contribute to less favorable therapeutic outcomes. Although such mechanisms provide biological plausibility for our findings, they were not directly investigated in this study and therefore should not be considered causal explanations for the observed associations.

It was observed that patients undergoing MD had significantly lower rates of microbiological confirmation compared to those not undergoing dialysis. This difference may be related to the higher prevalence of extrapulmonary TB in the dialysis group, since microbiological confirmation is often more difficult in these clinical presentations. The lower confirmation rate may increase the likelihood of incorrect diagnostic classifications, a fact to consider when interpreting the observed associations [27–30]. However, all cases included in the study met the notification criteria established by the Brazilian TB surveillance system [31].

In addition to a higher incidence, patients with CKD often present with atypical clinical pictures of TB. Extrapulmonary manifestations and nonspecific symptoms are more frequent, making diagnosis difficult [19, 20, 32, 33]. It was identified that patients on MD presented a higher frequency of pleural and lymph node involvement, reinforcing the clinical complexity of this group. This finding is aligned with previous studies indicating a higher proportion of extrapulmonary TB among individuals with advanced kidney disease [19, 20, 34, 35]. From a clinical standpoint, such presentations can lead to delayed diagnosis, reduced rates of microbiological confirmation, and increased diagnostic uncertainty, all of which can compromise treatment outcomes in this population. Meanwhile, studies with people in advanced CKD or on dialysis report higher mortality rates and lower treatment success, even in scenarios where cure rates are between 70% and 80% [20, 21]. Factors such as greater disease severity, the use of hemodialysis, hypoalbuminemia, and pulmonary tuberculosis itself have been consistently associated with worse prognoses [20, 21, 36]. Although there is a considerable volume of publications on the risk of TB and clinical characteristics in CKD, studies that robustly quantify treatment outcomes among patients receiving MD remain scarce, especially in contexts of high disease burden and resource constraints. From this perspective, our study refines this understanding by quantifying the impact specifically on treatment outcomes in a high-incidence setting.

The probabilistic linkage methodology enabled us to overcome common limitations of registry-based studies, such as underreporting and interoperability issues across databases. Furthermore, the literature reports potential biological mechanisms underlying increased susceptibility and worse outcomes in this population, highlighting immune dysfunction associated with uremia, chronic inflammation, and malnutrition [27, 37–43] However, it should be noted that these factors were not directly evaluated in the present study and, therefore, should not be considered a causal explanation for the observed associations.

A highly relevant and pragmatic finding of our study is the protective role of DOT, which was associated with a 33% reduction in the odds of unfavourable treatment outcomes (aOR 0.67). These findings are consistent with a significant body of evidence demonstrating that DOT reduces treatment failure, loss to follow-up, mortality, and the emergence of drug resistance compared with self-administered therapy [27, 44, 45]. Systematic reviews and meta-analyses reinforce these findings, reporting higher treatment success rates and lower loss to follow-up among patients receiving DOT [44, 46, 47–50]. These benefits extend even to multidrug-resistant TB, where maintaining DOT throughout therapy significantly increases the proportion of patients achieving favourable treatment outcomes (approximately 67% vs. 47%) [48]. From a policy perspective, dialysis clinics may represent strategic settings for DOT implementation. Patients receiving MD attend healthcare facilities several times per week, creating regular opportunities for treatment supervision, adherence support, symptom monitoring, and early identification of adverse events. In regions such as Amazonas, where geographic barriers and fragmented healthcare services may compromise continuity of care, greater integration between dialysis services and TB control programs could improve treatment adherence and reduce the risk of unfavourable treatment outcomes. These findings support the development of coordinated care models in which dialysis units function not only as providers of renal replacement therapy but also as important hubs for TB surveillance, treatment support, and integrated patient care.

Our analysis suggested an inverse association between diabetes mellitus and unfavourable TB treatment outcomes (aOR 0.84), which should be interpreted with considerable caution. This finding is inconsistent with the established body of evidence, which generally identifies diabetes as a risk factor for delayed sputum conversion, treatment failure, relapse, and mortality among individuals with TB [51–56]. Therefore, the observed association should not be interpreted as evidence of a biological protective effect of diabetes. Rather, it is more likely explained by methodological factors inherent to observational studies and the use of secondary data. Possible explanations include residual confounding, differential healthcare access, more frequent clinical follow-up, misclassification of comorbidities or outcomes, and selection mechanisms such as collider bias and survivor bias [57]. Individuals with diabetes, particularly those receiving dialysis or other forms of chronic care, are often more engaged with healthcare services through structured follow-up programs. This increased healthcare engagement may facilitate earlier diagnosis, closer clinical supervision, improved treatment adherence, and more complete ascertainment of outcomes. Evidence from integrated TB-diabetes care programs suggests that intensified monitoring and continuity of care can improve patient retention and treatment follow-up [52]. Consequently, the inverse association observed in our study may reflect patterns of healthcare utilization rather than a true causal protective effect. Furthermore, collider bias may have influenced the observed results. Since inclusion in healthcare pathways is affected by both diabetes status and factors associated with TB outcomes, conditioning access to healthcare services may distort the relationship between diabetes and treatment outcomes. These mechanisms suggest that the inverse association is more plausibly explained by access and selection processes for healthcare services than by a protective biological effect. Thus, this finding should be considered hypothesis-generating and interpreted as a likely artifact of the observational study design, and not as evidence supporting a protective role of diabetes in TB treatment outcomes.

Beyond clinical factors, social determinants played a crucial role in treatment outcomes. Illicit drug use, alcohol consumption, and smoking were associated with higher odds of unfavorable outcomes, reinforcing the idea that TB in the Brazilian Amazon is not only a biomedical challenge but also a reflection of social vulnerability and structural inequalities. These factors must be interpreted within the broader context of the Amazonian health system, where large geographical distances, dependence on river transport, the concentration of specialized services in Manaus, and difficulties in continuity of care can amplify the impact of social disadvantage on treatment adherence and follow-up [58–62]. Such barriers are particularly relevant for patients requiring specialized services, such as an MD, who often depend on coordinated care at multiple levels of the health system. Addressing TB, therefore, requires not only clinical interventions but also strategies that integrate social support, harm reduction, patient referral, and improved coordination between primary care, TB control programs, and specialized services.

Our findings confirm and expand upon the results of recent studies in Brazil, which highlight the strong relationship between adverse outcomes in tuberculosis treatment and factors such as social vulnerability, HIV infection, substance use, and difficulties in accessing healthcare services [3, 61–63]. Research from national cohorts shows that therapeutic success across the country remains influenced by regional inequalities and comorbidities, reinforcing the urgency of interventions targeting higher-risk populations [64–68]. Despite the scarcity of specific evidence on patients undergoing maintenance dialysis in the Brazilian context, our study advances the literature by identifying this condition as an independent predictor of unfavorable tuberculosis outcomes in the Brazilian Amazon, a region with a high incidence of the disease and considerable challenges in accessing medical care.

The strengths of this study include the large cohort size; the use of a unified dialysis registry covering virtually the entire network of renal replacement therapy centers in the state of Amazonas, with the exception of a single center that did not participate; and the robustness of the probabilistic record linkage, which enabled the identification and accurate temporal classification of MD cases within a population-based TB cohort.

Some limitations need to be considered in our study. The use of secondary data from surveillance systems restricted access to key clinical variables, such as residual renal function, nutritional status, and specific causes of death, limiting more detailed analyses. In addition, the identification of dialysis patients relied on data from private renal replacement therapy centers, which provide virtually all dialysis services in the state. These services are not fully integrated into routine public health surveillance systems, and their professionals are not consistently engaged in standardized reporting, training, or data system integration. As a result, important variables, particularly the date of MD initiation, were incompletely recorded, reducing data completeness and potentially affecting exposure classification. Although the participating clinics represent the major providers of renal replacement therapy in Amazonas, one dialysis center did not contribute data. Consequently, some patients receiving MD may not have been identified, potentially leading to exposure misclassification and underestimation of the number of exposed individuals.

Although MD accounted for a small fraction of the study population (0.2%), the observed association remained statistically consistent. However, the small number of individuals exposed (*n* = 112), coupled with incomplete dialysis modality records for a large proportion of patients, limited the feasibility of subgroup and sensitivity analyses, such as analyses of complete cases and stratification by dialysis modality. Consequently, it was not possible to formally assess the robustness of the results considering different analytical assumptions, which may have influenced the accuracy and generalizability of the estimated associations.

An additional limitation concerns the inverse association observed between diabetes mellitus and unfavorable treatment outcomes. This result, contradicting established literature, may reflect residual confounding, misclassification, differences in access to healthcare services, or selection mechanisms such as survivor bias and collider bias. Therefore, the identified association should not be interpreted as evidence of a protective causal effect of diabetes.

The classification of outcomes was based on routinely collected surveillance data. Information on the cause of death was not independently validated, and some transferred patients may have been incorrectly classified as dropouts if treatment outcomes were not recorded at the receiving unit. These factors may have introduced some error in the classification of outcomes.

We excluded 1,545 records (2.6%) due to missing information on treatment outcomes. Although this proportion is relatively small, the possibility of selection bias cannot be ruled out. Individuals with missing data may have differed systematically from those included in the analysis, for example, in disease severity, access to health services, or follow-up completeness. Since there was no information on outcomes in these records, it was not possible to formally assess the direction and magnitude of any resulting bias.

Patients undergoing MD had lower rates of microbiological confirmation than individuals not undergoing dialysis. In these cases, the diagnosis was more frequently based on clinical and epidemiological criteria, especially in the context of extrapulmonary TB, which may lead to some degree of misdiagnosis.

Although the probabilistic linkage procedures were carefully validated, including detailed manual review, a residual risk of misclassification or mismatched records cannot be excluded. Finally, as this is an observational study, causal relationships cannot be established. The mechanisms underlying the observed associations were not directly evaluated and should therefore be interpreted as hypotheses based on the available literature. Future prospective studies are needed to better clarify these pathways.

## Conclusion

In this large population-based TB cohort from the Brazilian Amazon, MD emerged as a rare but clinically important condition associated with poorer treatment outcomes, identifying a particularly vulnerable population within TB control programs. Our findings support greater integration between TB programs and dialysis services, including systematic TB screening, early diagnosis, treatment monitoring, and the implementation of patient-centred adherence interventions such as DOT. Given these patients’ regular contact with healthcare services, dialysis units are strategic settings for integrated TB care and treatment support. More broadly, these findings reinforce the importance of integrating TB care with the management of chronic non-communicable diseases to improve continuity of care and reduce adverse outcomes among high-risk populations, particularly in resource-constrained and geographically challenging settings such as the Brazilian Amazon.

Strengthening collaborative care models, expanding access to preventive therapy, and improving data integration between surveillance and specialized services are essential steps to mitigate the intersection between communicable and non-communicable diseases in high-burden contexts.

## Supplementary Information

Below is the link to the electronic supplementary material.

Supplementary Material 1

## Data Availability

The data that support the findings of this study are available from the corresponding author upon reasonable request.

## Declarations

### Statement

During the preparation of this work, the authors utilized ChatGPT (version 4, from OpenAI, San Francisco, California, USA) and Grammarly to refine and summarize the language. After using this tool/service, the authors reviewed and edited the content as needed and take full responsibility for the publication's content.

## Abbreviations

CIs: 95% Confidence intervals
CKD: Chronic kidney disease
DOT: Directly Observed Therapy
FVS-AM: Amazonas Health Surveillance Foundation
MD: Maintenance dialysis
IQRs: Interquartile ranges
ORs: Odds ratios
aORs: Adjusted odds ratios
PLWHA: People living with HIV/AIDS
TB: Tuberculosis
