# Supplementary material for: Association between maintenance dialysis and tuberculosis treatment outcomes in Amazonas, Brazil: a population-based record-linkage cohort study (2001–2023): Maintenance Dialysis and Tuberculosis Outcomes
Source: BMC Infect Dis. 2026 Jul 13;26:1781. doi: 10.1186/s12879-026-13991-3 (PMC13644016; doi:10.1186/s12879-026-13991-3)
Supplement: Supplementary file 1 [file 12879_2026_13991_MOESM1_ESM.docx]

**Supplementary Table S1.** Baseline characteristics of tuberculosis patients according to maintenance dialysis exposure status, Amazonas, Brazil, 2001–2023.

| **Characteristics** | **Study population**  **(No MD + MD)**  **(n = 58,611)** | **No maintenance**  **Dialysis (No MD)**  **(n = 58,499)** | M**aintenance dialysis (MD)**  **(n = 112)** | **P-value** |
| --- | --- | --- | --- | --- |
| Sex, male | 35458 (60.5%) | 35391 (60.5%) | 67 (59.8%) | 0.960 |
| Region, Manaus | 47559 (81.1%) | 47448 (81.1%) | 111 (99.1%) | <0.001 |
| Age, Years | 35.2 [24.1;51.1] | 35.2 [24.1;51.1] | 48.6 [34.7;58.0] | <0.001 |
| Race (non-white) | 48547 (82.8%) | 48452 (89.5%) | 95 (90.5%) | <0.001 |
| Zone, urban | 51386 (89.2%) | 51279 (87.7%) | 107 (95.5%) | 0.004 |
| Chest radiography suggestive of TB | 42690 (72.8%) | 42602 (72.8%) | 88 (78.6%) | 0.063 |
| Microbiologic diagnosis | 39550 (67.5%) | 39493 (67.5%) | 57 (50.9%) | <0.001 |
| Contacts | 3.0 [1.0;5.0] | 3.0 [1.0;5.0] | 2.0 [0.0;5.0] | <0.001 |
| TB type, pulmonary | 48875 (83.4%) | 48815 (83.4%) | 60 (53.6%) | <0.001 |
| Pleural or lymph node TB | 4538 (7.7%) | 4508 (7.7%) | 30 (26.8%) | <0.001 |
| PLWHA^a^ | 5487 (9.4%) | 5486 (9.4%) | 1 (0.9%) | 0.004 |
| CKD | 338 (0.6%) | 226 (0.4%) | 112 (100.0%) | <0.001 |
| CKD – before TB diagnosis | 204 (0.4%) | 92 (0.2%) | 112 (100.0%) | <0.001 |
| Diabetes Mellitus | 3977 (6.8%) | 3948 (6.7%) | 29 (25.9%) | <0.001 |
| Mental illness | 548 (0.9%) | 548 (0.9%) | 0 (0.0%) | 0.631 |
| Drug use | 2357 (4.0%) | 2354 (4.0%) | 3 (2.7%) | 0.632 |
| Smoking | 3493 (5.9%) | 3488 (6.0%) | 5 (4.5%) | 0.639 |
| Alcohol | 5144 (8.8%) | 5138 (8.8%) | 6 (5.4%) | 0.266 |
| DOT | 11100 (18.9%) | 11090 (19.0%) | 10 (8.9%) | 0.010 |
| Outcome (Unfavourable) | 15856 (27.1%) | 15783 (27.0%) | 73 (65.2%) | <0.001 |

**Source:** SINAN-TB/FVS-RCP; Registry of CKD cases – Maintenance Dialysis, Amazonas, Brazil, 2001–2023 (updated August 2024).

**Abbreviations:** TB, Tuberculosis; PLWHA, People living with HIV/AIDS; DOT, Directly Observed Treatment; CKD, Chronic Kidney Disease.

Notes:

**a** They represented 94.4% of all HIV infection cases.

Percentages were calculated using non-missing observations for each variable; therefore, denominators vary across variables owing to missing data. Percentages for race and zone are based on records with non-missing information (race, 92.6% complete; zone, 98.3% complete)
